# Effects of forces on chromatin

**DOI:** 10.1063/5.0065302

**Published:** 2021-10-13

**Authors:** Kshitij Amar, Fuxiang Wei, Junwei Chen, Ning Wang

**Affiliations:** 1 Department of Mechanical Science and Engineering, The Grainger College of Engineering, University of Illinois at Urbana-Champaign, Urbana, Illinois 61801, USA; 2 Key Laboratory of Molecular Biophysics of the Ministry of Education, Department of Biomedical Engineering, College of Life Science and Technology, Huazhong University of Science and Technology, Wuhan, Hubei 430074, China

## Abstract

Chromatin is a unique structure of DNA and histone proteins in the cell nucleus and the site of dynamic regulation of gene expression. Soluble factors are known to affect the chromatin structure and function via activating or inhibiting specific transcription factors. Forces on chromatin come from exogenous stresses on the cell surface and/or endogenous stresses, which are regulated by substrate mechanics, geometry, and topology. Forces on chromatin involve direct (via adhesion molecules, cytoskeleton, and the linker of nucleoskeleton and cytoskeleton complexes) and indirect (via diffusion and/or translocation processes) signaling pathways to modulate levels of chromatin folding and deformation to regulate transcription, which is controlled by histone modifications and depends on magnitude, direction, rate/frequency, duration, and modes of stresses. The rapid force transmission pathway activates multiple genes simultaneously, and the force may act like a “supertranscription factor.” The indirect mechanotransduction pathways and the rapid force transmission pathway together exert sustained impacts on the chromatin, the nucleus, and cell functions.

## I. INTRODUCTION

**FIG. 1.**
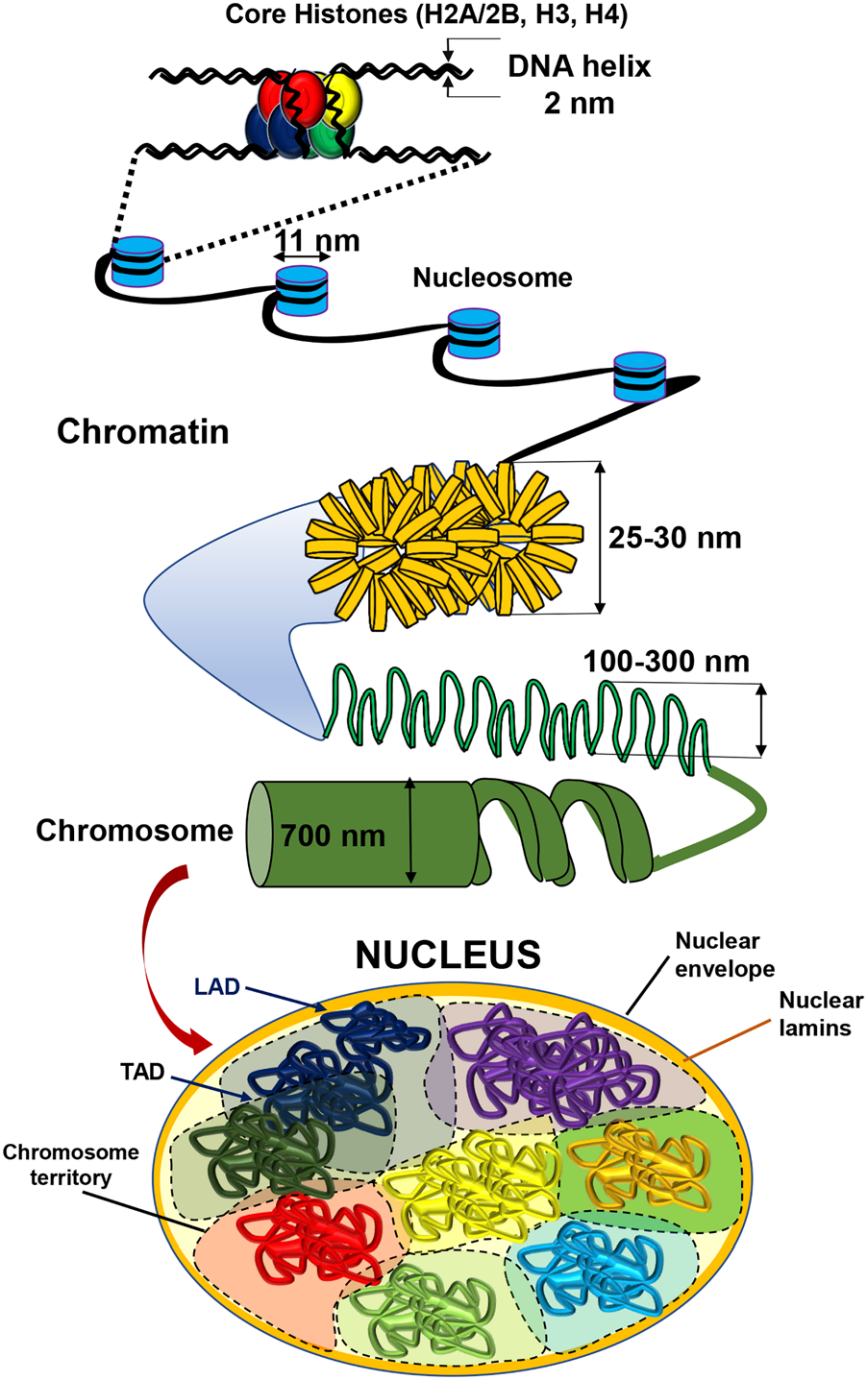
Schematic of the chromatin structure and the nucleus. DNA wraps around an octamer of histones (two H2A, two H2B, two H3, and two H4) to form a nucleosome and the chromatin fiber. The chromatin fiber is folded to organize into complex structures of chromatin domains. LAD, lamina-associated domain. TAD, topologically associated domain, i.e., chromatin domain, which associates with each other to create chromatin compartments. Chromosome territory, the DNA of a chromosome. During metaphase of cell division, the chromatins are condensed into higher orders of structures, chromosomes, which are detectable by conventional light microscopy. For brevity, other structures such as nucleolus and Cajal body and molecules in the nucleus are not illustrated.

Chromatin is a complex of DNA and histone proteins in the cell nucleus, the largest organelle inside a eukaryotic cell. A primary function of the chromatin is to package long molecules of DNA into compact and dense structures for dynamic regulation of gene transcription, DNA replication, and DNA repair. In order to fit into a small nuclear space of several micrometers in diameter, the DNA, a molecule of 2 nm diameter and hundreds of millimeters long, must wrap around histones and fold into compact and dense structures that range from the ∼10-nm “beads on a string” chromatin fibers and the 25 to 30-nm loops to the 100 to 300-nm chromatin domains and compartments^1^ (Fig. 1). It is well known that soluble transcription factors regulate gene expression by turning “on” or “off” genes. However, the compact state of chromatin often limits access of transcription factors and RNA polymerase II to DNA promoters. Moreover, histone modifications regulate chromatin domains for local transcription control.^2^

**FIG. 2.**
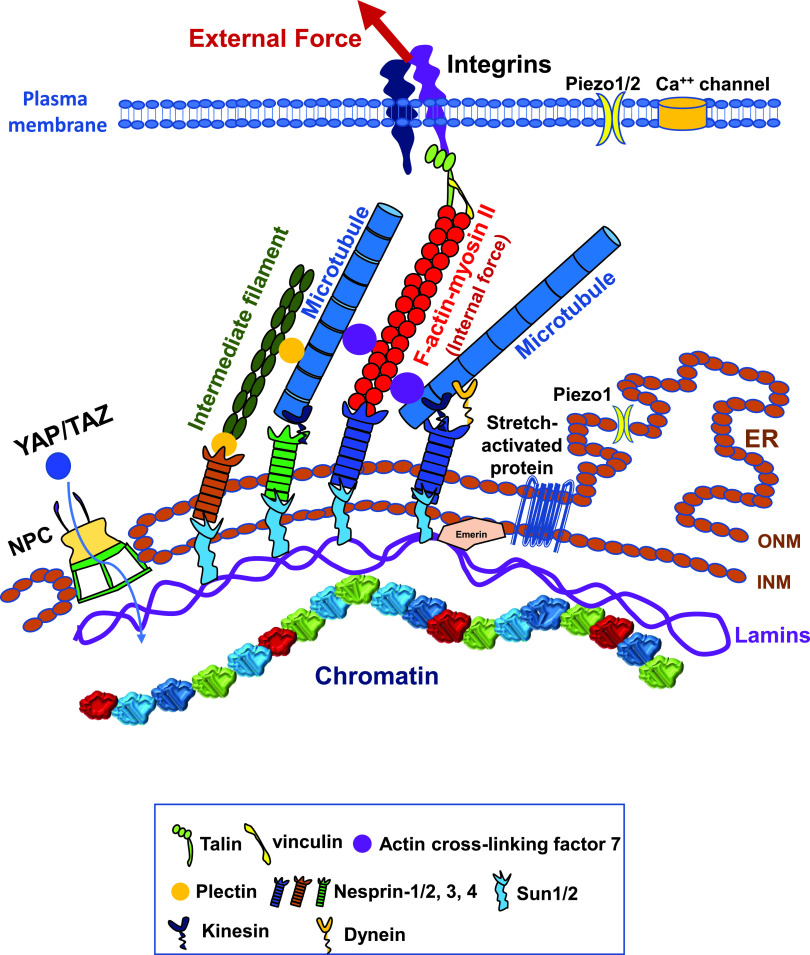
The force transmission pathway from the cell surface to the chromatin. External forces are transmitted from the extracellular matrix proteins to the integrins, intracellular focal adhesion proteins (talin and vinculin and other proteins), filamentous actin (F-actin) (which is associated with nonmuscle myosin II), and from F-actin to the LINC (linker of nucleoskeleton and cytoskeleton) complex (nesprins and Sun1/2), to the nuclear lamina networks, and then to the chromatin. ONM, outer nuclear membrane; INM, inner nuclear membrane; ER, endoplasmic reticulum. Plasma membrane deformation by the large force can also open Piezo1/2 mechanosensitive channels and stretch-activated calcium channels to signal or causes YAP/TAZ to translocate into the nucleus via the nuclear pores. Piezo1 on the endoplasmic reticulum can be activated to release intracellular calcium. Stretch-activated protein, a putative protein at the nuclear membranes which responds to mechanical stretch. For brevity, the force pathway via cell–cell adhesion molecules is not drawn.

In the early 2000s, proteins of the LINC (linker of nucleoskeleton and cytoskeleton) complex have been identified. The LINC complex consists of KASH-domain proteins across the outer nuclear membrane that link the cytoskeleton in the cytoplasm with SUN proteins across the inner nuclear membrane that connect to the nuclear lamina (Lamin A/C and Lamin B).^3,4^ These structural linkages from the cytoplasm to the nucleus, together with transmembrane molecule integrins that bind cytoplasmic focal adhesion proteins (e.g., talin and other structural proteins) and the actin cytoskeleton at the plasma membrane site and lamina-associated domains (LADs)^5^ of the chromatin that associate the nuclear lamina with the rest of the chromatin at the intranuclear side, offer a structural pathway for force transmission from the cell surface to the chromatin (Fig. 2). In this review, we highlight the effects of forces on chromatin and the ensuing changes in gene expression.

## II. EARLY EVIDENCE OF APPLIED STRESS TO DEFORM THE NUCLEUS AND CHROMATIN

**FIG. 3.**
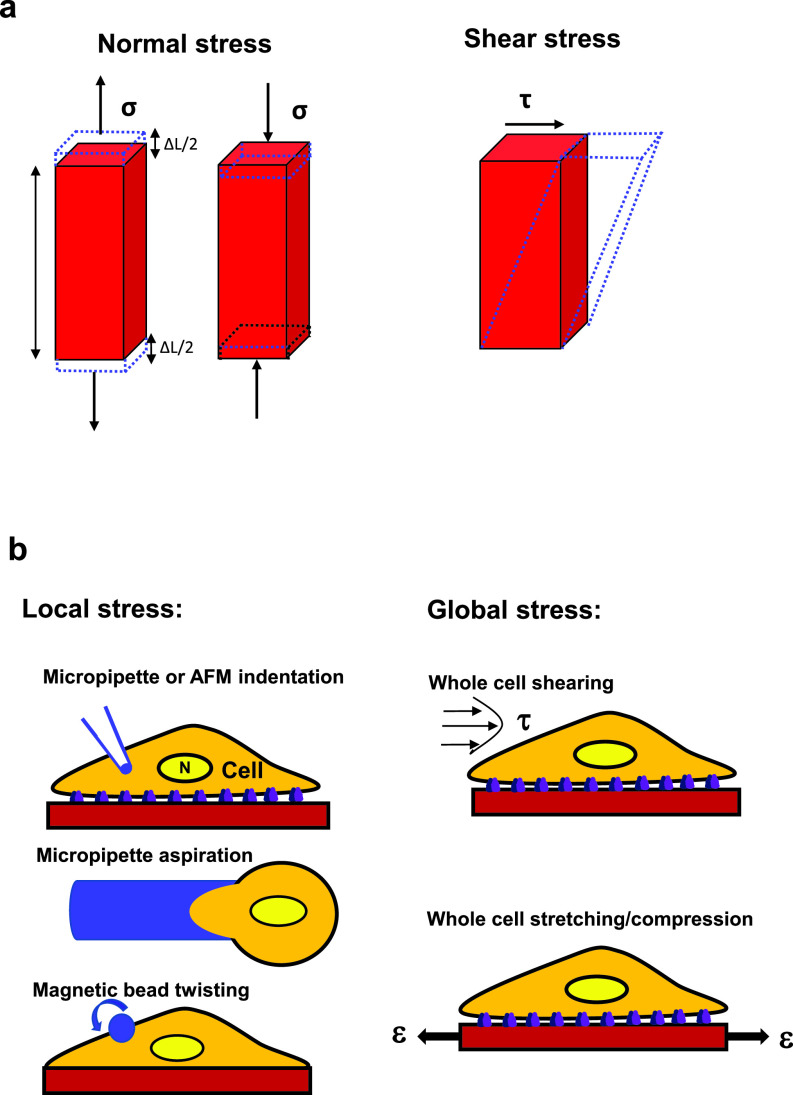
Impact of force on materials and the cell. (a) Normal stress σ (left; tensile or compressive) induces normal strain (dashed lines), ε = ΔL/L, where L is the original length and ΔL is the change in length; shear stress τ (right) induces the shear strain or shape change (dashed lines) of the material. Stress is defined as force per unit area. (b) Left, local stress: micropipette or AFM (atomic force microscopy) indentation, micropipette aspiration, or magnetic bead twisting. N, nucleus. Right, global stress: fluid shear stress over the whole cell surface or substrate stretching (tensile) strain ε (uniaxial or biaxial) (or substrate compression by reversing the direction of the applied strain ε) of the whole cell in the x–y plane. The whole cell can also be stretched or compressed along the vertical (z) direction using the parallel-plates rheometer or the optical stretcher.^140^ Note that the term “force” is often used generically, representing a mechanical load.

Increasing experimental evidence has demonstrated the effects of forces and mechanics on the cell nucleus. Since it is known that forces that are exerted on individual living cells and their substructures are dominated by surface interactions, the contact area of the force is important and the physiological relevant input that the cell responds to is stress, which is defined as force per unit area and has a unit of newton per square meter or pascal. Stress is sub-characterized into shear stress and normal stress (tensile stress or compressive stress). A tensile stress is applied in the same direction as the direction of the local surface area, and a compressive stress is applied in the opposite direction. Normal stress results in normal strain (strain is defined as deformation divided by original length and is dimensionless); shear stress, on the other hand, is applied tangential to the direction of the local surface area and results in shear strain and shape change (Fig. 3). An early report has shown that when a fibronectin-coated micropipette indentation is used to deform the endothelial cell surface (Fig. 3) by 10–20 *μ*m, it can cause nuclear deformation although the nucleus is ∼5–10-fold stiffer than the cytoplasm.^6^ However, in that study, the magnitude of cell surface deformation is comparable to the cell diameter (∼15 *μ*m) and the micropipette produces a much larger deformation than an endothelial cell generally experiences in blood vessels under physiological conditions. Therefore, it is not clear if a physiologically relevant small surface deformation can also deform the stiff nucleus. An alternative approach of using an RGD(Arg-Gly-Asp)-coated magnetic bead twisting (Fig. 3) to apply a physiologically relevant local stress of 10–20 Pa via integrins shows that the nucleolus inside the nucleus can be directly deformed.^7^ Since a blood flow-induced shear stress in blood vessels *in vivo* on the apical surface of an endothelial cell^8^ is ∼1–8 Pa and total focal adhesion areas at the basal surface are ∼20% of the cell apical surface area,^9^ from the force balance, one can determine that flow induced shear stress at the focal adhesions of an endothelial cell is fivefold higher and reaches 5–40 Pa, which is similar to the magnitude of the stress applied via magnetic bead twisting. A different approach is to use a micropipette aspiration pressure to suck the cell into the micropipette to deform the nucleus (Fig. 3). It is shown that Lamin A/C but not Lamin B contributes to nuclear stiffness.^10,11^ Micropipette aspiration of embryonic stem cells and adult stem cells shows that their nuclei (due to lack of Lamin A/C) are much softer than those of differentiated epithelial cells.^12^ In Lamin A/C deficient cells, under a large deformation induced by the micropipette aspiration, chromatin flows and reorganizes while the nuclear lamina stretches, suggesting that the nuclear lamina contributes to nuclear modulus and the chromatin and the nucleoplasm determine the viscous properties of the nucleus under these large deformation conditions.^12^ Nuclear viscoelasticity, quantified with magnetic nanorods, is decreased in Lamin A/C knockout mouse embryonic fibroblasts.^13^ These results suggest that the nucleus is a distinct mechanical structure from the cytoplasm and the nuclear lamin networks contribute to nuclear mechanics.

## III. MECHANICS OF ISOLATED NUCLEI AND CHROMOSOME

Because the nucleus lies inside the cytoplasm of the cell, it is rather difficult to gain access to the nucleus and to study its mechanics in a living cell, especially when the mechanical strain or the applied stress is small. An early study using a micropipette to deform a mitotic chromosome pulled out of a living cell finds that its Young's modulus is ∼1–5 kPa (Ref. 14) (1 kPa = 1000 Pa). In contrast, isolated chromosomes have a modulus of ∼0.3 kPa, lower than that of the chromosome pulled out of a living cell.^15^ However, when a 30-pN force is pulled directly to the nesprins on the nuclear envelope, the stiffness of an isolated nucleus is only ∼0.6 Pa,^16^ ∼3 orders of magnitude lower than that of the chromosome. It is possible that the applied tensile force to the isolated nucleus only deforms the nuclear envelope and not the underlying nuclear lamina. While some biological features, such as Emerin phosphorylation, are still retained in isolated nuclei in response to the applied pulling force, some important nuclear force-transmitting proteins might be lost when the nucleus is isolated from the living cell. A different method of an angular optical trap is used to measure torque in chromatin, and it is shown that a braided chromatin fiber is torsionally stiff and a single chromatin fiber that is a preferred substrate for topoisomerase II is torsionally soft.^17^ Magnetic tweezers have been utilized to probe the chromatin structure of reconstituted chromatin fibers, and higher-order structure of different chromatin fibers can be inferred by fitting a statistical mechanics model to the force-extension data.^18^ Monte Carlo simulations are used to mimic single-molecule force spectroscopy experiments that reveal different stages of chromatin fiber unfolding when the applied force ranges from 5 to 20 pN.^19^ Furthermore, atomic force microscopy (AFM) has been used for both an imaging tool and a force spectroscopy tool for isolated chromatin and DNA.^20^ For instance, a study using AFM (and optical tweezers) reveals that high mobility group B (HMGB) proteins strongly disrupt nucleosomes to regulate chromatin accessibility.^21^ Compared with the micropipette indentation approach in which the applied force or stress is difficult to quantify, the AFM indentation method has the advantage of precise tuning of cantilever modulus and probe size such that the applied force or stress can be quantified. While the studies on isolated chromatin fibers provide insights into the mechanisms of force impact, micropipette indentation and AFM indentation (Fig. 3) can also be used to study the effects of forces on chromatin in living cells. For example, a recent report using AFM indentation on a living cell reveals that after cisplatin-induced DNA damage nuclear stiffness is significantly reduced as a result of global chromatin decondensation.^22^ It will be helpful in future to combine the studies of isolated chromatin fibers with those in living cells to better understand the effects of forces in chromatin in physiological and pathological conditions.

## IV. EFFECTS OF SUBSTRATE MECHANICS AND GEOMETRY ON CHROMATIN

It is known for a while that substrate elasticity regulates cell fate by controlling gene expression patterns in adult stem cells.^23^ One report shows that phosphorylation and localization of chromatin modifying enzyme histone deacetylase are regulated by matrix stiffness to influence fibroblast to myofibroblast transition.^24^ Recently, it is reported that mesenchymal stem cells respond to matrix stiffening by increasing nuclear tension and histone acetylation via deactivation of histone deacetylases, committing to osteogenic fate.^25^ In contrast, elevated substrate stiffness leads to elevation in the activity of histone deacetylases that transforms fibroblasts into myofibroblasts and induces condensed chromatin.^26^ A “motor clutch” model is proposed to explain the molecular mechanism of substrate rigidity sensing via integrins,^27^ but this model might need to be modified since a recent report shows that myosin filaments generate much larger forces per motor in a cell than measured in single-molecule experiments.^28^ Nevertheless, it is well-accepted that myosin II and F-actin plays an important role in substrate rigidity sensing and stiff substrates activate myosin II to generate greater cytoskeletal stresses (prestress) in the cytoplasm. Since the force must be balanced, the myosin II dependent prestress near the cell surface also exerts its effect on size and shape of the nuclear envelope and the chromatin structure via the cytoskeleton and the LINC (linker of nucleoskeleton and cytoskeleton) complex. The substrate stiffness can exert irreversible impact on transcription factors in the stem cells when the substrate is switched from stiff to soft; in other words, the cells exhibit mechanical memory.^29^ When the cells are cultured on stiff substrates for a few weeks, microRNA (miRNA21) accumulates, which is critical in fibrotic mechanical memory of mesenchymal stem cells and is regulated via mechanosensitive myocardin-related transcription factor-A (MRTF-A) and remains elevated 2 weeks after mechanical stimulus cessation.^30^ This is consistent with the report that one week after induction of mesenchymal stem cells with matrix elasticity, the substrate starts to direct the cells toward substrate-stiffness specific lineage, and by 3 weeks, the matrix stiffness induced differentiation becomes committed and exhibits mechanical memory, which is unperturbed even in trans-induction media.^23^ Histone acetylation and chromatin organization are shown to adapt rapidly after substrate softening and are reversible or irreversible depending on the duration of the cells in stiff microenvironments,^31^ suggesting that epigenetic modifications are persistent and could play a key role in mechanical memory. The applied stress of ∼25 Pa elevates the level of histone 3 lysine 9 dimethylation/trimethylation (H3K9me2/3) after 60 min of load application.^32^ In addition, after switching the stem cell-like tumor-repopulating cells from soft (∼100 Pa) 3D fibrin matrix to 2D rigid plastic, it takes 2 days to upregulate H3K9me2/3 in the chromatin, suggesting that mechanical memory and plasticity may exist in soft stem-cell-like tumor-repopulating cells since the stiffening of these cells starts at 6 h and it takes 1 week for the stiff substrate to reach the stiffness values of differentiated tumor cells,^32^ although it is possible that 1 week merely reflects the time needed for the cells to differentiate; however, these two mechanisms are not mutually exclusive. Other long-term changes in the chromatin structure and the nuclear structure likely contribute to the mechanical memory as stem cells (or cancer stem cells) differentiate or differentiated cells are reprogrammed back to undifferentiated cells. The effect of substrate stiffness on cell fate and behavior has also been extended to substrate viscous behavior, independent of substrate elastic modulus, degradation, and cell-adhesion-ligand density.^33^ These studies demonstrate that substrate mechanics can critically regulate cell fate, function, and behaviors via altering the nucleus and the chromatin.

A geometric cue is generally considered as a different cue from substrate mechanics. Using micropatterned substrates to alter cellular geometry (shape, aspect ratio, and size), researchers show that different cell geometry leads to different gene-expression profiles, redistribution of histone deacetylase 3 modulated histone acetylation in an actomyosin-dependent manner.^34^ Experiments and modeling show that cell geometric constraints induce cytoskeleton-mediated nuclear lamina softening, chromatin stiffening, and nuclear lamina invaginations.^35^ Therefore, the underlying mechanism for cell geometry to influence chromatin structure and function includes alteration of distribution and alignment of the cytoskeleton and the anisotropy of actomyosin-dependent prestress, which, in turn, affects nuclear envelope shape, size, and location. In addition to flat surface geometry, the topology of the surface also impacts cell and nuclear structures and functions. For instance, a microgrooved substrate induces reorganization of the nuclear lamins and repositioning of chromosomes.^36^ Furthermore, microgrooved surfaces lead to increased histone H3 acetylation and methylation and promote mesenchymal-to-epithelial transition in adult fibroblasts.^37^ These microgrooves generate local plasma membrane distortion and cytoskeleton deformation to influence the nucleus and the chromatin. Together these findings suggest that geometry and topology regulate cell fate and function via altering the chromatin.

## V. MECHANICAL SIGNALING PATHWAYS

### A. Piezo1/2 and calcium signaling pathways

Since any mechanical perturbation or stimulation of living cells starts at the cell surface, it is logical for researchers to first examine what changes mechanical loading bring about at the plasma membrane and at the cytoplasmic side of the plasma membrane. One such structure that has received much attention is the transmembrane mechanosensitive ion channel. Piezo1/2 are mechanically activated cation channels,^38^ which are expressed in several cell types although the structure of channel opening has not been observed in a living cell. Piezo1 is required for embryonic vascular development in mice,^39^ and Piezo2 is required for Merkel cell mechanotransduction.^40^ However, the commonly used modes of mechanical stimulation of stretching the cell membrane include applying stresses via a patch-clamping electrode or a glass pipette. Hence, the magnitude of the applied stress has not been controlled precisely or measured accurately for Piezo1/2. In contrast, mechanosensitive ion channels (e.g., stretch-sensitive calcium channels) only open when the magnitude of the applied stress on an endothelial cell is >100 Pa (1–5 nN force on a 4.5-*μ*m diameter magnetic bead; 1 nN = 10^−9^ N).^41^ Follow-up studies show that the stress applied to β1 integrins leads to ultra-rapid (<4 ms) activation of calcium influx through transient receptor potential vanilloid 4 (TRPV4) ion channels,^42^ and the CD98hc (the transmembrane solute carrier family 3 member 2) protein is the key necessary adaptor protein^43^ that links β1 integrin with TRPV4 for rapid mechanotransduction at the cell surface. Moreover, cyclic stretch of mesenchymal stem cells for >2.5 min induces chromatin condensation via ATP (Adenosine triphosphate) release and purinergic calcium signaling dependent cytoskeletal contractility (prestress).^44^ However, calcium entry into the cell does not always have to rely on high stress or large-scale cell surface deformation. For example, a study that artificially elevates extracellular multivalent cations of living cells so that the extracellular calcium or magnesium level is 3–15-fold higher than the physiological level reveals that heterochromatin levels are increased through the activation of mechanosensitive ion channels in the absence of mechanical loading; the increased heterochromatin levels lead to upregulation of the nuclear stiffness.^45^ In contrast, stretching living cells triggers Piezo1-mediated cytoplasmic calcium release 30 s post-stretching from the intracellular calcium pool in the endoplasmic reticulum (Fig. 2); elevated cytoplasmic calcium is responsible for the decrease in H3K9me3 in the nucleus and subsequent nuclear softening and then the cellular response of the increase in perinuclear F-actin rings.^46^ Moreover, a recent report shows that the nucleus of a living cell that is compressed when the cell migrates through a confined matrix responds to the mechanical perturbation by stretching the nuclear envelope, leading to the activation of stretch-sensitive proteins at the nuclear envelope and potentially at the perinuclear endoplasmic reticulum to trigger the release of intracellular calcium and cytosolic phospholipase A2 (cPLA2) re-localization onto the stretched nuclear envelope and the increase in actomyosin contractility; and this response from the nucleus is independent of the extracellular calcium.^47^ In a separate report, it is shown that cell compression during confined migration leads to inner nuclear membrane unfolding that, in turn, triggers a calcium-dependent signaling via cPLA2 activation and arachidonic acid production to regualte myosin II activity; however, blocking tension-dependent Piezo1 channels using GsMTx4 in these cells do not reduce myosin II activity.^48^ The reason for the differences in the role of Piezo1 in cell stretching and cell confinement is not clear at this time but may be attributable to the different modes of mechanical deformation and/or the magnitude of deformation. In addition, some yet-to-be-identified stretch-sensitive proteins at the nuclear envelope may contribute to cell confinement induced cellular contraction responses. These studies show that the extracellular calcium and/or the intracellular calcium can be part of a signaling pathway for nuclear mechanotransduction under certain forcing conditions (Fig. 2).

### B. The YAP/TAZ pathway

About a decade ago, the cytoplasmic YAP/TAZ [Yes-associated protein/transcriptional coactivator with PDZ-binding motif; PDZ=Post synaptic density protein (PSD95), Drosophila disc large tumor suppressor, and Zonula occludens-1 protein] is found to function as a relay molecule that translocates into the nucleus when the cell is plated on stiff substrates.^49^ The translocation of YAP/TAZ is regulated by the actomyosin cytoskeletal prestress since increasing stress fibers and F-actin activates YAP/TAZ to enable it to translocate into the nucleus; focal adhesions [e.g., integrins, focal adhesion kinase (FAK), and Src, etc.] are also required for YAP/TAZ activity.^50^ A report finds that a compressive stress applied over the nucleus of a living cell triggers the nuclear entry of YAP by increasing YAP transport across nuclear pores.^51^ However, YAP/TAZ is not the only molecule whose translocation into the nucleus regulates gene transcription. Other cytoplasmic molecules such as MKL1 (megakaryoblastic leukemia 1)^52^ and Twist1^53^ are found to translocate into the nucleus independent of YAP/TAZ. For example, elevation of substrate stiffness promotes cytoplasmic Twist1 to translocate into the nucleus^53^ by releasing Twist1 from its binding partner G3BP2. In addition, the activation of YAP/TAZ is downstream from focal adhesions and the actin cytoskeleton such that YAP translocation into the nucleus takes many minutes to hours. Consistent with this signaling cascade is the report that YAP translocation from the cytoplasm into the nucleus occurs only after 4-h cyclic stretching of the flexible substrate.^54^ These findings suggest that the YAP/TAZ signaling pathway in response to mechanical stimulation is slower than the mechanosensitive ion channel pathway, responsible for generating sustained biological responses (Fig. 2).

### C. Integrin mechanosensing and integrin-mediated signaling

It is well established that integrin activation regulates cell adhesion and migration, cytoskeletal remodeling, extracellular matrix assembly, and mechanotransduction.^55^ Integrins are well-known force-sensing molecules.^56,57^ Focal adhesion proteins, such as talin and vinculin, are reported to be force-sensitive proteins downstream from integrins.^58,59^ Other filamentous actin associated proteins, such as filamin A^60^ and a-actinin,^61^ are also integrators of tension in the cell as well as focal adhesion proteins zyxin^62^ and paxillin.^63,64^ In fact, it is conceivable that spanning the mechanical linkages from integrins (or cell–cell mechanosensor cadherins^65^) to the chromatin, any force-bearing elements and their associated elements should be candidates of force-sensitive molecules. Hence, it is not surprising that actin filaments^66^ and myosin^67^ are demonstrated to be important force-sensitive elements in the cell. Even cell–cell adhesion adaptor protein α-catenin is able to detect filamentous actin tension.^68^ Up on attachment to immobilized extracellular matrix proteins, integrins sense force very rapidly (<0.5 s).^69^ One molecule in early integrin-mediated signaling is focal adhesion kinase (FAK),^70^ which regulates recruitments of cytoplasmic Src to focal adhesions. The crosstalk between integrins, Src, and Rho is known to regulate cell adhesion, spreading, and cytoskeletal reorganization.^71^ Early integrin-mediated cell adhesion results in the activation of Rac1 and Cdc 42 and inhibition of RhoA, leading to actin-mediated cell protrusion; subsequently, RhoA activity increases and Rac1/Cdc42 activity decreases, driving focal adhesion maturation and stress fiber formation.^71^ Mitogen-activated protein kinase (MAPK), which is downstream from FAK and Src, is also activated in integrin-mediated cell adhesion.^72^ During constrained cell migration, elevated Ras/MAPK signaling is associated with cytoskeleton rearrangements and cell softening.^73^ In addition, integrin-adhesion-induced MRTF-A (MKL1) activation and signaling promote cell migration and invasion,^74^ and MRTF-A/B suppresses ICAM-1 gene expression via forming a complex with NF-κB p65 in the nucleus.^75^ It is reported that on soft surfaces (0.3 kPa), myosin IIA deletion enhances ERK1/2 (a member of the MAPK family) activity and inhibits tumor cell invasion but enhances tumor cell proliferation; on hard surface (5 kPa), myosin IIA deletion increases NFκB activity and enhances tumor cell invasion.^76^ Together with the focal adhesion-regulated and substrate stiffness-mediated YAP/TAZ translocation, integrin-mediated MAPK, MRTF-A (MKL1), and NFκB signaling and their translocation into the nucleus appear to play important roles in regulating nuclear and chromatin activities and functions.

### D. The force transmission pathway of integrin–cytoskeleton–LINC complex

It is now well-known that a locally applied stress via integrins propagates to cytoplasmic focal adhesion proteins and F-actin, from F-actin (and other cytoskeletal filaments) to nesprins across the outer nuclear membrane, and then to Sun proteins across the inner nuclear membrane and to the nuclear lamina, from the lamina to the chromatin^4^ (Fig. 2). This mechanical and structural pathway provides a direct linkage from the extracellular matrix proteins to the chromatin. However, it is known from St. Venant's principle, any locally applied stress decays rapidly as a function of the square of the distance in any homogenous material. If this were the case, then the nucleus, which resides inside the cytoplasm and is several micrometers away from the local surface load, would not be able to feel the impact of the stress since the magnitude of the applied stress would become minuscule before it reaches the nucleus. An adherent and spread cell is anything but homogenous. Due to focal adhesions and prestressed actin bundles (i.e., stress fibers), the locally applied stress is concentrated along the stiff stress fibers and can be propagated to distances of tens of micrometers away from the local stress source to activate cytoplasmic enzymes and to deform the nucleus.^7,77–81^ The long distance stress propagation depends on the integrity of the stress fibers and is regulated by the cytoskeletal prestress.^7,77–82^ With this unique feature of stress concentration (stress focusing), it is possible to directly deform the stiff nucleus and the chromatin to elicit nuclear mechanotransduction such as gene expression without triggering activation by calcium or translocation of YAP/TAZ. Next, we discuss different ways of deforming a living cell and its chromatin and the consequences.

## VI. EFFECTS OF STRESSES ON CHROMATIN AND GENE EXPRESSION

### A. Stresses applied to the whole cell surface

#### 1. Shear stress

Living cells in the human body experience various forms of stresses: tensile stress, compressive stress, and shear stress (Fig. 3). An early report reveals the identification of a fluid shear-stress-responsive element at the platelet-derived growth factor (PDGF) B chain promoter site after the application of a laminar shear stress for several hours on the apical surface of the whole endothelial cell (1.0 Pa for 4 h).^83^ Indeed fluid shear stress applied to the apical surface of the whole cell (Fig. 3) from laminar, disturbed, or oscillatory flows generates different gene expression patterns and different responses from endothelial cells.^84^ The cell nucleus stiffens when endothelial cells are exposed to fluid shear stress.^85^ Fluid shear stress enhances the human embryonic stem cell priming via H2B acetylation and chromatin decondensation.^86^ It has been shown that differences between shear stress and normal stress on chromatin organization and gene expression apply to other types of cells besides endothelial cells (see Sec. VI C 3). These studies provide useful information on regulation of gene expression by fluid shear stress. However, the limitation is that gene expressions are generally measured tens of minutes to hours after stress application, so it is difficult to dissect out the exact underlying mechanisms and early mechanotranduction pathways responsible for the activation of the chromatin and early alterations of gene expression.

#### 2. Tensile stress

In contrast to the fluid shear stress, blood vessel pressure oscillations cause the whole endothelial cell to stretch. Other cell types in living tissues also experience substantial tensile stresses via extracellular matrices (Fig. 3). These stresses generate normal strains and shear strains in the cells and trigger a number of different responses in the endothelial cells in comparison to shear stress.^87^ Stretching of the whole cell by 3% at 1 Hz for >2.5 min (up to 180 min) leads to chromatin condensation via extracellular calcium signaling dependent actomyosin contractility.^44^ In contrast, stretching the cell monolayer by 5%–40% at 0.1 Hz for 30 min results in downregulation of H3K9me3 levels and nuclear softening (measured by AFM indentation) via intracellular calcium signaling;^46^ reorientation of stress fibers to the perpendicular direction from the stretching direction occurs at 360 min by 40% strain only.^46^ It is not clear what accounts for the difference in the status of the chromatin structure in these two studies. These findings suggest that tensile stresses impact the nucleus and the chromatin differently from shear stresses.

#### 3. Compressive stress

Numerous studies have shown that compressive stresses applied to the whole cells induce changes in gene expression.^88–91^ Compressive stresses of 1–2 kPa on the whole cell deform the nuclear envelope to activate ATR (Ataxia telangiectasia and Rad3 related) so that it translocates to the nuclear envelope, which influences the temporal process of chromatin condensation, presumably protecting the chromatin integrity.^92^ A later report demonstrates that a static compressive stress of 1 kPa for 1 h on the whole cell disrupts apical stress fibers and reduces cytoskeletal prestress to trigger translocation of histone deacetylase 3 (HDAC3) into the nucleus, leading to the elevation of H3K9me3 and H3K27me3 levels, condensation of chromatins, and alteration of gene transcription.^93^ However, a static 40% nuclear compression^46^ for 30 min results in a decrease in H3K9me3 levels. The reason for this discrepancy in compression-induced changes in H3K9me3 levels is not clear at this time; it is possible that part of the reason is due to cell plating density: the cells are geometrically constrained single cells in the HDAC3 translocation study and the cells form a confluent monolayer in the latter case. A recent study shows that a living cell responds to nuclear compression by elevating actomyosin contractility such that that cell quickly migrates away from the confinement site.^47^ Apico-basal cell compression of Drosophila epithelial tissues or of mammalian epithelial cells regulates the levels of Lamin A/C by modulating the phosphorylation of Lamin A/C at Serine 22, a target for Lamin A/C degradation.^94^ Tumor cells soften their nuclei to facilitate transendothelial extravasation,^95^ suggesting large deformation of the nucleus and the chromatin. Together these studies show that compressive stresses on the whole cell can exert their effects on the nucleus and alter the structure of chromatins via signaling cascades in the cytoplasm.

### B. Magnitude and duration of whole cell loading

#### 1. Small deformation vs large deformation

While the majority of physiologically relevant stresses causes only relatively small deformation (<10%) on the whole cell, in some cases, the deformation can be large (>20%). For example, when a migrating cell move through dense extracellular matrices, the cell must squeeze through narrow spaces during stem cell migration in embryonic development or cancer cell invasion. Under these physiological conditions or pathological conditions, as the whole cell is deformed, the nucleus and the chromatin are also deformed, resulting in nuclear envelope rupture and DNA damage.^96–101^ The nuclear lamina protects the cell from the nuclear envelope rupture and DNA damage,^102^ and mutant lamins can cause nuclear damage and skeletal muscle cell death.^103^ In some cases, transient (<0.2 s) but high (∼5 kPa) tensile stress on the nuclear envelope can cause nuclear membrane rupture.^104^ Recently, it is shown that a 40% uniaxial stretch of a monolayer of epidermal progenitor cells for 0.5 h causes softening of the nucleus to protect against DNA damage.^46^ In addition these studies demonstrate that under high stresses or large deformations that are applied externally or generated endogenously as the cells move through constricted pores, the nuclear envelope rupture occurs, which results in DNA damage.

#### 2. Short load duration vs long load duration

In the living body under physiological conditions, the effect of soluble molecules such as cytokines or growth factors is short-lived because of the diffusion-based dilution of the released molecules. The impact of the forces, however, can be long-lasting since all living cells generate contractile forces all the time. To simulate this process, researchers have applied mechanical stresses or deformation to living cells or tissues from minutes to hours to days. The cells respond to the duration of the applied stresses. For example, stretching the whole cell monolayer for 30 min at 40% stretch causes the deformation of the nucleus and the chromatin and a decrease in H3K9me3 in the nucleus.^46^ Extending the duration of stretching to 6 h leads to remodeling of the cytoskeleton such that stress fibers re-orient perpendicular to the stretch direction.^46^ Blocking the calcium release from the endoplasmic reticulum inhibits the 30-min response but not the stress fiber reorientation, suggesting that the short term (30 min) response is controlled by a different mechanism (i.e., intracellular calcium) from the long term (6 h) response. It is reported that exposing human epidermal stem cells to biaxial cyclic strain of 10% at 0.1 Hz for 3–12 h leads to emerin-enrichment dependent actomyosin mediated switching from H3K9me2/3 to H3K27me3 at constitutive heterochromatin,^105^ suggesting that applied strains alter H3K9me2/3 only after several hours of application. These published reports highlight the importance of stress duration for a given stress magnitude and rate in the structure and function of the nucleus and the chromatin.

### C. Stresses applied locally to the cell

Under physiological conditions, any living adherent cell experiences a local stress via cell–matrix adhesion molecule integrins from the extracellular matrix and/or via cell–cell adhesion molecules from other cells; in contrast, cells in suspension (e.g., blood cells) experience fluid shear stress and other forms of stresses via cell–cell contacts (e.g., between immune cells, platelets, and circulating tumor cells). As discussed earlier, a local stress via integrins could be transmitted via the cytoskeleton into the nucleus via the LINC complex to exert ultra-rapid deformation on the nuclear lamins and the chromatin (Fig. 2). As expected, defects or mutations in any of the proteins along this mechanical stress transmission pathway should potentially alter the structure and the mechanics of the nucleus and the chromatin. For example, deficiency in nuclear lamin A/C results in defects in nuclear mechanics,^106^ nuclear envelope rupture, and DNA damage in skeletal muscle cells.^103^ Depletion of nesprins in mammary epithelial cells leads to destabilization of the acinus.^107^ Depletion of nesprin-1 also abolishes reorientation of endothelial cells in response to cyclic strain.^108^ Disruption of the LINC complex (via depletion of nesprins or SUN proteins) impairs microneedle-caused stress transmission between the cytoskeleton (perinuclear actin and intermediate filaments) and the nuclear envelope, reduces nuclear deformation, and impairs nuclear positioning and cell polarization in migrating cells and in cells plated on micropatterned substrates.^109^ However, no inhibitory effects were observed in cyclic-strain-induced *Egr-1* and *Iex-1* gene expression after disruption of the LINC complex,^109^ possibly because the duration of stretching is long (30 min) such that indirect mechanotransduction (i.e., LINC complex-independent) pathways are activated to increase *Egr-1* and *Iex-1* gene expression and because the biaxial cyclic strain (5% at 1 Hz)^109^ is not large enough to reach the chromatin strain threshold to directly activate these two genes.

**FIG. 4.**
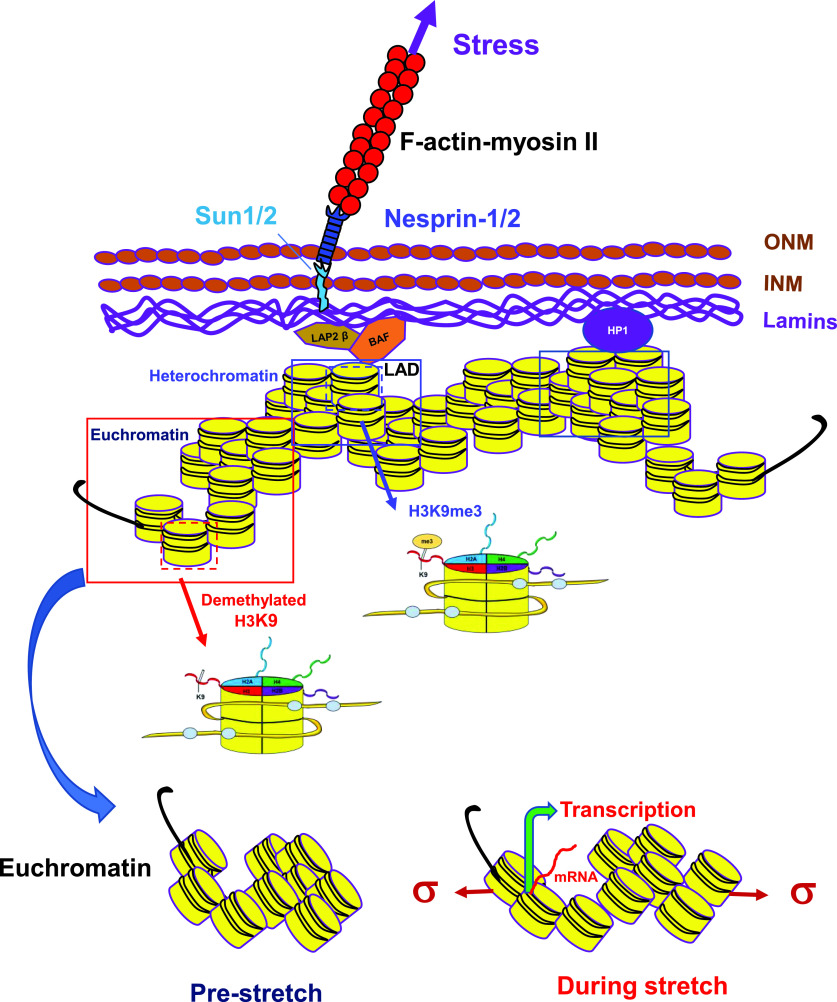
Stretching chromatin rapidly activates transcription at euchromatin domains. Externally applied stress (local or global on the cell surface) can directly stretch the chromatin domains via F-actin-myosin II, LINC, the nuclear lamina, and BAF (barrier-to-autointegration factor), possibly via LAP2β (lamina-associated polypeptide 2 beta), a putative force-transmitting molecule, and rapidly activate or upregulate transcription at the euchromatin sites away from the nuclear periphery that are demethylated at H3K9. Genes located at heterochromatin domains near the nuclear periphery, which are methylated at H3K9me3, cannot be rapidly activated by the applied stress. For visual clarity and simplicity, folded chromatin fibers are drawn to illustrate the chromatin architecture, including fibers, loops, and domains. ONM, outer nuclear membrane; INM, inner nuclear membrane; HP1, heterochromatin protein 1; LAD, lamina-associated domain; σ, stretching (tensile) stress.

In order to examine if the exogenous stress has a direct effect on the chromatin, one needs to quantify the extent of chromatin deformation. However, for years, it has been difficult to quantify deformation of the chromatin domain in a single chromatin because of the lack of probes and tools. Using a GFP labeled chromatin domain of transgene *DHFR* (dihydrofolate reductase, an essential molecule for the synthesis of thymine),^110^ it is demonstrated that the chromatin can be directly stretched by a local stress (6–15 Pa at 0.3–1.0 Hz, physiologically relevant magnitudes and frequencies) applied via a RGD-coated magnetic bead to the integrins and the extent of gene upregulation is tightly associated with the extent of chromatin stretching^111^ (Fig. 4). Remarkably, *DHFR* upregulation is dependent on the magnetic bead rolling angle relative to the cell long axis and the initiation of chromatin deformation and of the ensuing gene upregulation is within tens of milliseconds of stress application, suggesting that the gene is activated directly by chromatin stretching without relaying molecules as a result of cytoplasmic biochemical signaling cascades.^111^ BAF (barrier-to-autointegration factor) is an important structural protein^112^ that helps in transmitting the stress from the nuclear lamina to the chromatin since knocking down BAF inhibits external stress-induced gene upregulation^111^ (Fig. 4). Because of its structural associations with the nuclear lamina^113^ and with BAF,^114^ nuclear protein LAP2β is a putative molecule to mediate force transmission from the nuclear lamina to the chromatin, but the experimental evidence is still lacking (Fig. 4). A follow-up study reveals that endogenous genes *egr-1* (early growth response-1) and *Cav1* (calveolin-1) are also rapidly activated by a local stress via integrins after RNA polymerase II recruitment to their promoter sites;^115^ since upregulation of these two endogenous genes also depends on the magnetic bead stress angle relative to the cell long axis for the same magnitude of stress,^115^ these results likely rule out the contribution from the biochemical signaling cascades of the slow diffusive or translocation molecules. Importantly, the stress-induced gene activation depends on the chromatin domain demethylation at H3K9me3; H3K9me3 is high near the nuclear periphery at heterochromatin domains and H3K9me3 is low at euchromatin domain sites away from the nuclear periphery (Fig. 4). For a typical mechano-non-responsive gene *FKBP5* near the nuclear envelope in the heterochromatin region, when H3K9me3 is pharmacologically demethylated, this gene becomes activated by the exogenous stress as RNA polymerase II is recruited to its promoter site.^115^ These findings suggest that for the similar extent of stretching of the chromatin, the demethylation levels of H3K9me3 control which genes are activated, as the same stretching (tensile strain) on the chromatin is not able to activate genes as long as the chromatin domains are highly methylated at H3K9me3. The permissive effect of H3K9me3 demethylation also applies to gene expression in response to non-mechanical stimuli.^116^ It is possible that when the applied stress or strain is large and the stress duration is long (say, >tens of minutes), the levels of H3K9me3 can be modulated by external stress.^31^ Because the locally applied stress is able to rapidly upregulate or activate several genes simultaneously via deforming the chromatin, the finding suggests that the physiologically relevant stress acts like a supertranscriptional factor. The notion of the “supermechano-transcriptional factor” is supported by the observation that when human tumor cells are switched from 2D rigid substrate (modulus of ∼1 GPa, i.e., 1 × 10^9^ Pa) to 3D soft substrate (modulus of ∼100 Pa), many genes and molecules, including ATF4, SLC3A2, CCT3, and hsa-miR-199a-5p, are activated or upregulated to promote stemness and proliferation of tumor-repopulating cells.^117^ These malignant tumor-repopulating cells are a small subpopulation of cancer cells and are soft and metastatic.^118^ These cells express high levels of self-renewing gene Sox2 that is regulated by H3K9 methylation in the chromatin and are undifferentiated^118^ and resist conventional anti-cancer drug treatment.^118,119^ Under hypoxic conditions, reactive oxygen species are activated, leading to breast tumor-repopulating cell proliferation through metabolic reprogramming, which is absent in differentiated breast tumor cells.^120^ Recently, it is reported that cycling cancer persister cells that resist drug treatment are a small subpopulation of cancer cells and are dependent on oxidative stress or metabolic reprogramming for proliferation.^121^ It will be interesting to determine if the tumor-repopulating cells and the cycling cancer persister cells are a similar type of tumor cells arisen from a common precursor lineage or different lineages.

#### 1. Effects of force frequency on the chromatin

Cellular moduli are known to scale with the loading frequency in a weak-power law manner.^122^ However, it has been unclear how chromatin deformation is associated with the frequency of applied stresses. Using a RGD-coated magnetic bead to apply stresses of sinusoidal waveforms locally to integrins of cells, it is revealed that chromatin strains of *DHFR*-containing domains vary little between 0.3 and 6.0 Hz of loading but decrease dramatically at 20 Hz (Ref. 115) and, hence, not inversely proportional to the loading frequency on a log –log scale; in other words, the chromatin strains do not follow the weak-power law. This is likely due to the fact that the applied stresses concentrate along the actin bundles to propagate to long distances^82^ such that the stress in the chromatin decays slowly as the loading frequency increases. Importantly, transcription of transgene *DHFR* or endogenous genes *egr-1* and *Cav1* is rapidly upregulated in response to the cyclic stresses at low frequencies (0.3–20 Hz) but not at 100 Hz.^115^ These findings show that gene upregulation does not follow the weak-power law either. The lack of gene upregulation at 100 Hz does not appear to be due to the diminishing chromatin strains since elevating the applied stress magnitudes such that chromatin strains at 100 Hz exceed those at 20 Hz is still unable to upregulate the gene transcription.^115^ At 100 Hz, no RNA polymerase II is recruited to the promoter sites of the gene,^115^ suggesting that the likely underlying mechanism is the rapid unfolding and folding of chromatin domains (<5 ms at 100 Hz) that is too fast for RNA polymerase II to bind to the promoter sites of the gene.

#### 2. Direct vs indirect force impacts on the chromatin

It is possible that force sensitive elements at the cell surface of the cytoskeleton respond to mechanical forces in a direction-, amplitude-, frequency-, and duration-dependent manner by releasing/modifying signaling molecules, e.g., phosphorylating some transcription factors that then translocate to the nucleus since the cytoskeleton is anisotropic and responds to force directions. The finding that nuclear protein BAF depletion alone can completely abolish the effects of force directions on chromatin deformation and the ensuing gene upregulation^111^ suggests that this intranuclear molecule is a force-transmitting molecule, independent of the force-sensitive elements at the cell surface of the cytoskeleton. However, it is not clear, at this time, whether BAF depletion desensitizes the force-sensitive elements at the cell surface of the cytoskeleton, leading to blocking signaling molecules-mediated chromatin deformation. Therefore, this piece of evidence alone does not rule out the indirect mechanism. Another key piece of evidence supporting the mechanism from a direct force-dependent response of the nucleus comes from the very short time it takes to deform the chromatin when a force (should be stress to be precise) is applied at the cell surface via integrins. It takes only tens of milliseconds of time from the application of the stress on the cell surface to the deformation of the chromatin, suggesting the force is rapidly transmitted to the chromatin. More importantly, when the loading frequency is varied from 0.3 to 100 Hz, at 20 Hz of loading, there is substantial chromatin deformation and the ensuing gene upregulation.^115^ This result shows that the chromatin is deformed within 12.5 ms (one quarter of a sinusoidal stress cycle) of loading and this deformation is sufficient to activate gene transcription. Even at 100 Hz, stress-induced chromatin deformation is observed within 1–2 ms.^115^ It would be extremely difficult to interpret these results by the biochemical cascades and the signaling molecules in the cytoplasm initiated by the force sensitive elements at the cell surface of the cytoskeleton, even when one takes into account of the rapid diffusion or translocation rate of molecules. However, it is still possible that some force-sensitive elements in the cytoplasm that might be close enough to the nuclear membrane to mediate ultra-fast diffusion- or translocation-dependent mechanism might trigger the indirect mechanism of chromatin deformation. At this time, the indirect mechanism cannot be ruled out completely. Future work is needed to differentiate the direct from the indirect force-sensing mechanisms at the chromatin.

#### 3. Effects of force directions on the chromatin

It is well known that shear stresses at the endothelial cell apical surface produce different signaling and cellular responses than do stretching stresses at the base or sides of the cells.^123^ However, the mechanisms of how chromatin deformation and gene expression are altered by force directions have been elusive. It is reported in hamster ovary cells that local shear stresses (i.e., in-plane stresses that are in the plane of the cell apical surface) result in lower cell stiffness than normal stresses (i.e., out-of-plane stresses that are dominated by normal stresses) that lead to bead rolling along the cell long axis (0° angle, i.e., along the alignment of most actin stress fibers) or at different angles (90° or 45°) to the cell long axis.^82^ Chromatin stretching and ensuing *DHFR* gene upregulation by the shear stress mode are distinct from those induced by the 90°- or 0°-normal stress mode but similar to those by the 45°-normal stress mode.^82^ Theoretical finite element analysis modeling using discrete anisotropic stress fibers recapitulates the single cells experimental results and reveals that the underlying mechanism of force-direction dependence is due to the complex stress distribution in the stress fibers such that the shear stress and the 45°-normal stress elicit the same stress fiber strain patterns at the nuclear edge and, thus, the same chromatin deformation and the same ensuing gene transcription.^82^ These findings suggest that shear stress and normal stress at the cell surface elicit distinct chromatin organization changes and gene expression. It is important to point out that no matter what stress mode (normal stress or shear stress) it might be at the cell surface, the surface stress will generate both normal strains and shear strains on the chromatin because of the complex cytoskeletal organization and orientation. At the site of the chromatin domain that contains the gene of interest, it is the local tensile strain but not the shear strain that is the most critical to open up enough space to unfold the chromatin domain for RNA polymerase II to bind to upregulate gene transcription^82,111^ (Fig. 4).

Additionally, cytoskeletal prestress regulates the gene activation and upregulation as inhibition of myosin-II dependent prestress with myosin light chain kinase inhibitor ML-7 decreases stress-induced gene upregulation.^111^ Furthermore, pretreatment with myosin II ATPase inhibitor blebbstatin abrogates the difference in anisotropic gene upregulation by an out-of-plane stress (mostly normal stress) and by an in-plane stress (mostly shear stress) applied via integrins,^82^ indicating control of stress-induced anisotropic gene transcription by the cytoskeletal prestress. All these findings suggest that cytoskeletal prestress, a cellular hallmark in mechanobiology,^124^ plays a critical role in regulating chromatin stretching and rapid nuclear mechanotransduction and gene transcription.

## VII. STRESSES ON OTHER NUCLEAR STRUCTURES

It is well known that there are numerous membraneless sub-organelles inside the nucleus, such as the nucleolus,^125^ the site of ribosome synthesis, and the Cajal body,^126^ the site of spliceosome synthesis. Similar to the formation of self-organizing chromatin architecture^1^ that is driven by polymer–polymer interactions and liquid–liquid phase separation (LLPS), the formation of these membraneless nuclear structures is also suggested to originate from LLPS.^127^ The biomolecular condensates of LLPS can further transition to liquids, gels, or solids, depending on local conditions and properties of the condensates.^128^ There is evidence that the nucleolus is a multiphase liquid condensate.^129^ Phosphorylation of the RNA polymerase II C-terminal domain can drive a switch from transcription initiation condensates to RNA processing condensates.^130^ The initial condensation of the heterochromatin appears to depend on HP1 (heterochromatin protein 1) mediated liquid droplet formation of the heterochromatin domain.^131,132^ However, depletion of HP1 leads to elevated chromatin spontaneous movements and inhibition of cell surface stress-induced chromatin stretching and ensuing gene upregulation,^111^ suggesting that the HP1-dependent heterochromatin domain at the nuclear lamina behaves as a solid to transmit force (Fig. 4), which is consistent with a recent finding that both heterochromatin and euchromatin in living cells behave as solid-like scaffolds.^133^ Another example is that Fus protein fibrils form a hydrogel that, in turn, can be dissolved back into a liquid by DNA-dependent protein kinase.^134^ Molecular dynamics simulations show that nucleosome plasticity is critical in regulating chromatin LLPS.^135^ However, a review on the issue of LLPS concludes that the evidence for LLPS in live cells is phenomenological, and the causal relationship and functional consequences of LLPS in cells are still elusive.^136^ Those researchers suggest to use the term “hub” instead of LLPS to describe the formation of transient high concentration of molecules.^136^ An early work using a magnetic bead bound to integrins shows that the membraneless nucleolus in living cells can be directly deformed by a local stress with a physiological magnitude of 10–20 Pa and both 2D bulk strains (dilatational and compressive strains) and shear strains are induced by the stress, although the bulk strains dominate.^7^ These findings suggest that the membraneless nucleolus, whether or not it is formed via LLPS or via other mechanisms, behaves as a hydrogel under physiological conditions. A later work on another intranuclear membraneless structure Cajal body demonstrates direct dissociation of coilin-SMN (survival motor neuron) protein complexes within the Cajal body by a local stress applied via integrins.^137^ Dissociation of coilin-SMN is quantified with the fluorescence resonance energy transfer (FRET) probe and depends on force magnitude, an intact F-actin, Lamin A/C, substrate rigidity, and importantly by cytoskeletal prestress.^137^ Other protein pairs in the Cajal body exhibit different magnitudes of dissociation from the coilin-SMN complex for the same local cell surface stress. A step-function stress induces an almost step deformation response in the FRET signal between SMN and coilin and between other protein–protein pairs in the Cajal body,^137^ suggesting that these protein complexes behave like an elastic material or a viscoelastic hydrogel. The membraneless nuclear speckle is shown to amplify gene expression by the mechanism of gene-speckle association leading to decreased nascent transcript degradation by exosomes.^138^ The movements of genes within the chromatin domain toward the nuclear speckles are long-range (>0.5 *μ*m) and directional,^138^ suggesting again that the chromatin is solid-like hydrogels. Although whether the nuclear speckle behaves as a gel or a liquid remains to be determined, the nuclear speckle elongates in the direction of its movement,^138^ suggesting that it may behave as a gel-like structure. Together these findings suggest that while phase separation or other mechanisms may trigger the formation of biomolecular condensates, intrinsically disordered regions, or hubs of membraneless structures, the chromatins and other sub-organelles in the nucleus behave as hydrogel-like or sold-like scaffolds to bear and to transmit stresses while soluble molecules move into and out of these scaffolds.

A model of structural proteins (e.g., lamin A, myosin II, collagen, etc.) in the physical pathway of tension shows that stresses inhibit degradation of the structural proteins to achieve stable “mechanosensitive” gene expression if a given structural protein positively regulates its own gene expression.^139^ This “use-it-or-lose-it” model illustrates the importance of stresses in suppressing enzyme-mediated/initiated structural protein degradation. Currently, it is not clear if the endogenous stress and/or the applied stress will facilitate or inhibit the disassembly of the protein condensates or the protein hubs, but it is likely that the magnitude, duration, mode (tensile, shear, or compressive or rotational), and frequency of the stress all play important roles in regulating protein aggregation or degradation.

### A. Challenges and outlooks

Increasing evidence shows that exogenous forces or endogenous forces of the cell play a critical role in regulating the structure and function of the chromatin. Because the stresses are vectors and can be long-lasting, the impact of stresses on the nucleus and the chromatin is substantially distinct from that of soluble factors. The direct and rapid force transmission pathway can activate multiple genes simultaneously and may act like a supertranscription factor, and together with the indirect mechanotransduction pathway exerts sustained impacts on cell functions. The changes in the nucleus and chromatin structure by mechanics and by stresses have profound implications in human diseases and pathology (see other reviews in this special issue). For example, it was recently reported that substrate-rigidity triggered nuclear mechanosensing drives remodeling of chromatins such that the chromatin of myofibroblasts from patients with aortic valve stenosis exhibits a more condensed structure than that of myofibroblasts from healthy donors.^26^ While the outlook for mechanoregulation of chromatins looks exciting and vibrant, many challenges are faced in the field of mechanobiology. A major challenge is the inaccessibility of the chromatin in a living cell for direct intervention and manipulation. Another challenge is the complexity of the chromatin architecture such that the real-time readout of biochemical and biological activities and functions of the chromatin in a single cell is difficult. To study mechanoresponsitivity of the chromatin structure and function of a single cell in a living tissue or organism represents additional challenges for researchers in mechanobiology and mechanomedicine. For example, it is not known at present how forces applied to the membraneless structures in the nucleus might alter their activities to impact chromatin organization and gene transcription. It is also not clear how the interplay between genetic alterations and specific epigenetic modifications might regulate nuclear activities and functions. In the future, the potential roles of various intranuclear proteins or molecules in scaffolding, signaling, chromatin tethering, or force transmission need to be elucidated too. Novel force-based approaches and technologies^140^ that are combined with genetic manipulations, together with soluble-factor based manipulations and super-resolution imaging techniques, await researchers to develop and to utilize to understand the intricacies of the chromatin, the hub of transcription and DNA replication.

## Data Availability

The data that support the findings and the conclusions of this study are referenced and available within the article.

## References

[1] T. Misteli , Cell 183, 28–45 (2020). 10.1016/j.cell.2020.09.014 32976797 PMC7541718

[2] A. J. Bannister and T. Kouzarides , Cell Res. 21, 381–395 (2011). 10.1038/cr.2011.22 21321607 PMC3193420

[3] N. Wang , J. D. Tytell , and D. E. Ingber , Nat. Rev. Mol. Cell Biol. 10, 75–82 (2009). 10.1038/nrm2594 19197334

[4] T. J. Kirby and J. Lammerding , Nat. Cell Biol. 20, 373 (2018). 10.1038/s41556-018-0038-y 29467443 PMC6440800

[5] B. van Steensel and A. S. Belmont , Cell 169, 780 (2017). 10.1016/j.cell.2017.04.022 28525751 PMC5532494

[6] A. J. Maniotis , C. S. Chen , and D. E. Ingber , Proc. Natl. Acad. Sci. U. S. A. 94, 849 (1997). 10.1073/pnas.94.3.849 9023345 PMC19602

[7] S. H. Hu , J. X. Chen , J. P. Butler , and N. Wang , Biochem. Biophys. Res. Commun. 329, 423 (2005). 10.1016/j.bbrc.2005.02.026 15737604

[8] B. J. Ballermann , A. Dardik , E. Eng , and A. Liu , Kidney Int. Suppl. 67, S100 (1998). 10.1046/j.1523-1755.1998.06720.x 9736263

[9] P. F. Davies , A. Robotewskyj , and M. L. Griem , J. Clin. Invest. 91, 2640 (1993). 10.1172/JCI116503 8514872 PMC443328

[10] J. Lammerding , P. C. Schulze , T. Takahashi , S. Kozlov , T. Sullivan , R. D. Kamm , C. L. Stewart , and R. T. Lee , J. Clin. Invest. 113, 370 (2004). 10.1172/JCI200419670 14755334 PMC324542

[11] J. Lammerding , L. G. Fong , J. Y. Ji , K. Reue , C. L. Stewart , S. G. Young , and R. T. Lee , J. Biol. Chem. 281, 25768 (2006). 10.1074/jbc.M513511200 16825190

[12] J. D. Pajerowski , K. N. Dahl , F. L. Zhong , P. J. Sammak , and D. E. Discher , Proc. Natl. Acad. Sci. U. S. A. 104, 15619 (2007). 10.1073/pnas.0702576104 17893336 PMC2000408

[13] A. Celedon , C. M. Hale , and D. Wirtz , Biophys. J. 101, 1880 (2011). 10.1016/j.bpj.2011.09.008 22004741 PMC3192970

[14] B. Houchmandzadeh , J. F. Marko , D. Chatenay , and A. Libchaber , J. Cell Biol. 139, 1–12 (1997). 10.1083/jcb.139.1.1 9314524 PMC2139812

[15] M. G. Poirier , S. Eroglu , and J. F. Marko , Mol. Biol. Cell 13, 2170 (2002). 10.1091/mbc.01-08-0401 12058078 PMC117633

[16] C. Guilluy , L. D. Osborne , L. Van Landeghem , L. Sharek , R. Superfine , R. Garcia-Mata , and K. Burridge , Nat. Cell Biol. 16, 376 (2014). 10.1038/ncb2927 24609268 PMC4085695

[17] T. T. Le , X. Gao , S. H. Park , J. Lee , J. T. Inman , J. H. Lee , J. L. Killian , R. P. Badman , J. M. Berger , and M. D. Wang , Cell 179, 619 (2019). 10.1016/j.cell.2019.09.034 31626768 PMC6899335

[18] A. Kaczmarczyk , T. B. Brouwer , C. Pham , N. H. Dekker , and J. van Noort , Methods Mol. Biol. 1814, 297 (2018). 10.1007/978-1-4939-8591-3 29956240

[19] B. E. de Jong , T. B. Brouwer , A. Kaczmarczyk , B. Visscher , and J. van Noort , Biophys. J. 115, 1848 (2018). 10.1016/j.bpj.2018.10.007 30366627 PMC6303278

[20] D. P. Melters and Y. Dalal , J. Mol. Biol. 433, 166720 (2021). 10.1016/j.jmb.2020.11.019 33221335 PMC8770095

[21] M. J. McCauley , R. Huo , N. Becker , M. N. Holte , U. M. Muthurajan , I. Rouzina , K. Luger , L. J. Maher III , N. E. IsraeloffE , and M. C. Williams , Nucleic Acids Res. 47, 666 (2019). 10.1093/nar/gky1119 30445475 PMC6344895

[22] A. Dos Santos , A. W. Cook , R. E. Gough , M. Schilling , N. A. Olszok , I. Brown , L. Wang , J. Aaron , M. L. Martin-Fernandez , F. Rehfeldt , and C. P. Toseland , Nucleic Acids Res. 49, 340 (2021). 10.1093/nar/gkaa1202 33330932 PMC7797048

[23] A. J. Engler , S. Sen , H. L. Sweeney , and D. E. Discher , Cell 126, 677 (2006). 10.1016/j.cell.2006.06.044 16923388

[24] Y. Li , C. B. Tang , and K. A. Kilian , Cell. Mol. Bioeng. 10, 405 (2017). 10.1007/s12195-017-0493-8 31719870 PMC6816600

[25] A. R. Killaars , C. J. Walker , and K. S. Anseth , Proc. Natl. Acad. Sci. U. S. A. 17, 21258 (2020). 10.1073/pnas.2006765117 PMC7474590 32817542

[26] C. J. Walker , C. Crocini , D. Ramirez , A. R. Killaars , J. C. Grim , B. A. Aguado , K. Clark , M. A. Allen , R. D. Dowell , L. A. Leinwand , and K. S. Anseth , Nat. Biomed. Eng. (published online) (2021). 10.1038/s41551-021-00709-w

[27] C. E. Chan and D. J. Odde , Science 322, 1687 (2008). 10.1126/science.1163595 19074349

[28] J. Lohner , J. F. Rupprecht , J. Hu , N. Mandriota , M. Saxena , D. P. de Araujo , J. Hone , O. Sahin , J. Prost , and M. P. Sheetz , Nat. Phys. 15, 689 (2019). 10.1038/s41567-019-0477-9 33790983 PMC8008990

[29] C. Yang , M. W. Tibbitt , L. Basta , and K. S. Anseth , Nat. Mater. 13, 645 (2014). 10.1038/nmat3889 24633344 PMC4031270

[30] C. X. Li , N. P. Talele , S. Boo , A. Koehler , E. W. Knee , J. L. Balestrini , P. Speight , A. Kapus , and B. Hinz , Nat. Mater. 16, 379 (2017). 10.1038/nmat4780 27798620

[31] A. R. Killaars , J. C. Grim , C. J. Walker , E. A. Hushka , T. E. Brown , and K. S. Anseth , Adv. Sci. 6, 1801483 (2018). 10.1002/advs.201801483 PMC6364489 30775233

[32] Y. Tan , A. Tajik , J. Chen , Q. Jia , F. Chowdhury , L. Wang , J. Chen , S. Zhang , Y. Hong , H. Yi , D. C. Wu , Y. Zhang , F. Wei , Y. C. Poh , J. Seong , R. Singh , L. J. Lin , S. Doğanay , Y. Li , H. Jia , T. Ha , Y. Wang , B. Huang , and N. Wang , Nat. Commun. 5, 4619 (2014). 10.1038/ncomms5619 25099074 PMC4133791

[33] O. Chaudhuri , L. Gu , D. Klumpers , M. Darnell , S. A. Bencherif , J. C. Weaver , N. Huebsch , H. P. Lee , E. Lippens , G. N. Duda , and D. J. Mooney , Nat. Mater. 15, 326 (2016). 10.1038/nmat4489 26618884 PMC4767627

[34] N. Jain , K. V. Iyer , A. Kumar , and G. V. Shivashankar , Proc. Natl. Acad. Sci. U. S. A. 110, 11349 (2013). 10.1073/pnas.1300801110 23798429 PMC3710882

[35] F. Alisafaei , D. S. Jokhun , G. V. Shivashankar , and V. B. Shenoy , Proc. Natl. Acad. Sci. U. S. A. 116, 13200 (2019). 10.1073/pnas.1902035116 31209017 PMC6613080

[36] L. E. McNamara , R. Burchmore , M. O. Riehle , P. Herzyk , M. J. Biggs , C. D. Wilkinson , A. S. Curtis , and M. J. Dalby , Biomaterials 33, 2835 (2012). 10.1016/j.biomaterials.2011.11.047 22248989

[37] T. L. Downing , J. Soto , C. Morez , T. Houssin , A. Fritz , F. Yuan , J. Chu , S. Patel , D. V. Schaffer , and S. Li , Nat. Mater. 12, 1154 (2013). 10.1038/nmat3777 24141451 PMC9675045

[38] B. Coste , J. Mathur , M. Schmidt , T. J. Earley , S. Ranade , M. J. Petrus , A. E. Dubin , and A. Patapoutian , Science 330, 55–60 (2010). 10.1126/science.1193270 20813920 PMC3062430

[39] S. S. Ranade , Z. Qiu , S. H. Woo , S. S. Hur , S. E. Murthy , S. M. Cahalan , J. Xu , J. Mathur , M. Bandell , B. Coste , Y. S. Li , S. Chien , and A. Patapoutian , Proc. Natl. Acad. Sci. U. S. A. 111, 10347 (2014). 10.1073/pnas.1409233111 24958852 PMC4104881

[40] S. H. Woo , S. Ranade , A. D. Weyer , A. E. Dubin , Y. Baba , Z. Qiu , M. Petrus , T. Miyamoto , K. Reddy , E. A. Lumpkin , C. L. Stucky , and A. Patapoutian , Nature 509, 622 (2014). 10.1038/nature13251 24717433 PMC4039622

[41] B. D. Matthews , D. R. Overby , R. Mannix , and D. E. Ingber , J. Cell Sci. 119, 508 (2006). 10.1242/jcs.02760 16443749

[42] B. D. Matthews , C. K. Thodeti , J. D. Tytell , A. Mammoto , D. R. Overby , and D. E. Ingber , Integr. Biol. 2, 435 (2010). 10.1039/c0ib00034e PMC3147167 20725677

[43] R. Potla , M. Hirano-Kobayashi , H. Wu , H. Chen , A. Mammoto , B. D. Matthews , and D. E. Ingber , J. Cell Sci. 133, jcs248823 (2020). 10.1242/jcs.248823 32989042 PMC7657480

[44] S. J. Heo , S. D. Thorpe , T. P. Driscoll , R. L. Duncan , D. A. Lee , and R. L. Mauck , Sci. Rep. 5, 16895 (2015). 10.1038/srep16895 26592929 PMC4655352

[45] A. D. Stephens , P. Z. Liu , V. Kandula , H. Chen , L. M. Almassalha , C. Herman , V. Backman , T. O'Halloran , S. A. Adam , R. D. Goldman , E. J. Banigan , and J. F. Marko , Mol. Biol. Cell 30, 2320 (2019). 10.1091/mbc.E19-05-0286 31365328 PMC6743459

[46] M. M. Nava , Y. A. Miroshnikova , L. C. Biggs , D. B. Whitefield , F. Metge , J. Boucas , H. Vihinen , E. Jokitalo , X. P. Li , J. M. G. Arcos , B. Hoffmann , R. Merkel , C. M. Niessen , K. N. Dahl , and S. A. Wickstrom , Cell 181, 800 (2020). 10.1016/j.cell.2020.03.052 32302590 PMC7237863

[47] A. J. Lomakin , C. J. Cattin , D. Cuvelier , Z. Alraies , M. Molina , G. P. F. Nader , N. Srivastava , P. J. Saez , J. M. Garcia-Arcos , I. Y. Zhitnyak , A. Bhargava , M. K. Driscoll , E. S. Welf , R. Fiolka , R. J. Petrie , N. S. De Silva , J. M. Gonzalez-Granado , N. Manel , A. M. Lennon-Dumenil , D. J. Mueller , and M. Piel , Science 370, eaba2894 (2020). 10.1126/science.aba2894 33060332 PMC8059074

[48] V. Venturini , F. Pezzano , F. Catala Castro , H.-M. Hakkinen , S. Jimenez-Delgado , M. Colomer-Rosell , M. Marro , Q. Tolosa-Ramon , S. Paz-Lopez , M. A. Valverde , J. Weghuber , P. Loza-Alvarez , M. Krieg , S. Wieser , and V. Ruprecht , Science 370, eaba2644 (2020). 10.1126/science.aba2644 33060331

[49] S. Dupont , L. Morsut , M. Aragona , E. Enzo , S. Giulitti , M. Cordenonsi , F. Zanconato , J. L. Digabel , M. Forcato , S. Bicciato , N. Elvassore , and S. Piccolo , Nature 474, 179 (2011). 10.1038/nature10137 21654799

[50] A. Totaro , T. Panciera , and S. Piccolo , Nat. Cell Biol. 20, 888 (2018). 10.1038/s41556-018-0142-z 30050119 PMC6186418

[51] A. Elosegui-Artola , I. Andreu , A. E. M. Beedle , A. Lezamiz , M. Uroz , A. J. Kosmalska , R. Oria , J. Z. Kechagia , P. Rico-Lastres , A.-L. L. Roux , C. M. Shanahan , X. Trepat , D. Navajas , S. Garcia-Manyes , and P. Roca-Cusachs , Cell 171, 1397 (2017). 10.1016/j.cell.2017.10.008 29107331

[52] C. Y. Ho , D. E. Jaalouk , M. K. Vartiainen , and J. Lammerding , Nature 497, 507 (2013). 10.1038/nature12105 23644458 PMC3666313

[53] S. C. Wei , L. Fattet , J. H. Tsai , Y. Guo , V. H. Pai , H. E. Majeski , A. C. Chen , R. L. Sah , S. S. Taylor , A. J. Engler , and J. Yang , Nat. Cell Biol. 17, 678 (2015). 10.1038/ncb3157 25893917 PMC4452027

[54] Y. Cui , F. M. Hameed , B. Yang , K. Lee , C. Q. Pan , S. Park , and M. Sheetz , Nat. Commun. 6, 6333 (2015). 10.1038/ncomms7333 25704457 PMC4346610

[55] S. J. Shattil , C. Kim , and M. H. Ginsberg , Nat. Rev. Mol. Cell Biol. 11, 288 (2010). 10.1038/nrm2871 20308986 PMC3929966

[56] N. Wang , J. P. Butler , and D. E. Ingber , Science 260, 1124 (1993). 10.1126/science.7684161 7684161

[57] B. Geiger , J. P. Spatz , and A. D. Bershadsky , Nat. Rev. Mol. Cell Biol. 10, 21–33 (2009). 10.1038/nrm2593 19197329

[58] A. del Rio , R. Perez-Jimenez , R. Liu , P. Roca-Cusachs , J. M. Fernandez , and M. P. Sheetz , Science 323, 638 (2009). 10.1126/science.1162912 19179532 PMC9339221

[59] C. Grashoff , B. D. Hoffman , M. D. Brenner , R. Zhou , M. Parsons , M. T. Yang , M. A. McLean , S. G. Sligar , C. S. Chen , T. Ha , and M. A. Schwartz , Nature 466, 263 (2010). 10.1038/nature09198 20613844 PMC2901888

[60] A. J. Ehrlicher , F. Nakamura , J. H. Hartwig , D. A. Weitz , and T. P. Stossel , Nature 478, 260 (2011). 10.1038/nature10430 21926999 PMC3204864

[61] P. W. Oakes , Y. Beckham , J. Stricker , and M. L. Gardel , J. Cell Biol. 196, 363 (2012). 10.1083/jcb.201107042 22291038 PMC3275371

[62] J. S. Cheah , K. A. Jacobs , T. W. Lai , R. Caballelo , J. L. Yee , S. Ueda , V. Heinrich , and S. Yamada , Mol. Biol. Cell. 32, 1221 (2021). 10.1091/mbc.E19-10-0568 33909446 PMC8351546

[63] S. Sen , M. Tewari , A. Zajac , E. Barton , H. L. Sweeney , and D. E. Discher , Eur. J. Cell Biol. 90, 249 (2011). 10.1016/j.ejcb.2010.06.005 20663583 PMC2970638

[64] D. W. Zhou , T. T. Lee , S. Weng , J. Fu , and A. J. García , Mol. Biol. Cell. 28, 1901 (2017). 10.1091/mbc.e17-02-0116 28468976 PMC5541841

[65] Q. le Duc , Q. Shi , I. Blonk , A. Sonnenberg , N. Wang , D. Leckband , and J. de Rooij , J. Cell Biol. 189, 1107 (2010). 10.1083/jcb.201001149 20584916 PMC2894457

[66] V. E. Galkin , A. Orlova , and E. H. Egelman , Curr. Biol. 22, R96 (2012). 10.1016/j.cub.2011.12.010 22321312 PMC3277726

[67] A. Mentes , A. Huehn , X. Liu , A. Zwolak , R. Dominguez , H. Shuman , E. M. Ostap , and C. V. Sindelar , Proc. Natl. Acad. Sci. U. S. A. 115, 1292 (2018). 10.1073/pnas.1718316115 29358376 PMC5819444

[68] L. Mei , S. Espinosa de Los Reyes , M. J. Reynolds , R. Leicher , S. Liu , and G. M. Alushin , eLife 9, e62514 (2020). 10.7554/eLife.62514 32969337 PMC7588232

[69] N. Strohmeyer , M. Bharadwaj , M. Costell , R. Fässler , and D. J. Müller , Nat. Mater. 16, 1262 (2017). 10.1038/nmat5023 29115292

[70] A. Howe , A. E. Aplin , S. K. Alahari , and R. L. Juliano , Curr. Opin. Cell Biol. 10, 220 (1998). 10.1016/S0955-0674(98)80144-0 9561846

[71] S. Huveneers and E. H. Danen , J. Cell Sci. 122, 1059 (2009). 10.1242/jcs.039446 19339545

[72] Q. Chen , M. S. Kinch , T. H. Lin , K. Burridge , and R. L. Juliano , J. Biol. Chem. 269, 26602 (1994). 10.1016/S0021-9258(18)47058-5 7929388

[73] D. A. Rudzka , G. Spennati , D. J. McGarry , Y. H. Chim , M. Neilson , A. Ptak , J. Munro , G. Kalna , A. Hedley , D. Moralli , C. Green , S. Mason , K. Blyth , M. Mullin , H. Yin , and M. F. Olson , J. Cell Sci. 132, jcs224071 (2019). 10.1242/jcs.224071 31152052 PMC6589089

[74] M. R. Hermann , M. Jakobson , G. P. Colo , E. Rognoni , M. Jakobson , C. Kupatt , G. Posern , and R. Fässler , J. Cell Sci. 129, 1391 (2016). 10.1242/jcs.177592 26872785

[75] H. S. Picariello , R. S. Kenchappa , V. Rai , J. F. Crish , A. Dovas , K. Pogoda , M. McMahon , E. S. Bell , U. Chandrasekharan , A. Luu , R. West , J. Lammerding , P. Canoll , D. J. Odde , P. A. Janmey , T. Egelhoff , and S. S. Rosenfeld , Proc. Natl. Acad. Sci. U. S. A. 116, 15550 (2019). 10.1073/pnas.1902847116 31235578 PMC6681735

[76] K. Hayashi , T. Murai , H. Oikawa , T. Masuda , K. Kimura , S. Muehlich , R. Prywes , and T. Morita , Sci. Rep. 5, 10627 (2015). 10.1038/srep10627 26024305 PMC4448521

[77] S. H. Hu , J. X. Chen , B. Fabry , Y. Numaguchi , A. Gouldstone , D. E. Ingber , J. J. Fredberg , J. P. Butler , and N. Wang , Am. J. Physiol.: Cell Physiol. 285, C1082 (2003). 10.1152/ajpcell.00159.2003 12839836

[78] S. H. Hu , L. Eberhard , J. X. Chen , J. C. Love , J. P. Butler , J. J. Fredberg , G. M. Whitesides , and N. Wang , Am. J. Physiol.: Cell Physiol. 287, C1184 (2004). 10.1152/ajpcell.00224.2004 15213058

[79] N. Wang and Z. G. Suo , Biochem. Biophys. Res. Commun. 328, 1133 (2005). 10.1016/j.bbrc.2005.01.070 15707995

[80] S. Na , O. Collin , F. Chowdhury , B. Tay , M. Ouyang , Y. Wang , and N. Wang , Proc. Natl. Acad. Sci. U. S. A. 105, 6626 (2008). 10.1073/pnas.0711704105 18456839 PMC2373315

[81] Y.-C. Poh , S. Na , F. Chowdhury , M. Ouyang , Y. Wang , and N. Wang , PLoS One 4, e7886 (2009). 10.1371/journal.pone.0007886 19924282 PMC2773925

[82] F. Wei , X. Xu , C. Zhang , Y. Liao , B. Ji , and N. Wang , Nat. Commun. 11, 4902 (2020). 10.1038/s41467-020-18584-5 32994402 PMC7524734

[83] N. Resnick , T. Collins , W. Atkinson , D. T. Bonthron , C. F. Dewey , and M. A. Gimbrone , Proc. Natl. Acad. Sci. U. S. A. 90, 4591 (1993). 10.1073/pnas.90.10.4591 8506304 PMC46558

[84] Y. S. J. Li , J. H. Haga , and S. Chien , J. Biomech. 38, 1949 (2005). 10.1016/j.jbiomech.2004.09.030 16084198

[85] S. Deguchi , K. Maeda , T. Ohashi , and M. Sato , J. Biomech. 38, 1751 (2005). 10.1016/j.jbiomech.2005.06.003 16005465

[86] J. Wang , Y. Wu , X. Zhang , F. Zhang , D. Lü , B. Shangguan , Y. Gao , and M. Long , Stem Cell Res. Ther. 10, 349 (2019). 10.1186/s13287-019-1454-z 31775893 PMC6880446

[87] J. Ando and K. Yamamoto , Antioxid. Redox Signaling 15, 1389 (2011). 10.1089/ars.2010.3361 20854012

[88] Y.-H. Lee , D.-S. Nahm , Y.-K. Jung , J.-Y. Choi , S. G. Kim , M. Cho , M.-H. Kim , C.-H. Chae , and S.-G. Kim , J. Periodontol. 78, 446 (2007). 10.1902/jop.2007.060240 17335367

[89] A. C. Shieh and K. A. Athanasiou , Osteoarthritis Cartilage 15, 328 (2007). 10.1016/j.joca.2006.08.013 17045815

[90] M. L. Berre , J. Aubertin , and M. Piel , Integr. Biol. 4, 1406 (2012). 10.1039/c2ib20056b 23038068

[91] T. Kanazawa , G. Nakagami , T. Minematsu , T. Yamane , L. Huang , Y. Mugita , H. Noguchi , T. Mori , and H. Sanada , PLoS One 9, e104676 (2014). 10.1371/journal.pone.0104676 25102054 PMC4125229

[92] A. Kumar , M. Mazzanti , M. Mistrik , M. Kosar , G. V. Beznoussenko , A. A. Mironov , M. Garre , D. Parazzoli , G. V. Shivashankar , G. Scita , J. Bartek , and M. Foiani , Cell 158, 633 (2014). 10.1016/j.cell.2014.05.046 25083873 PMC4121522

[93] K. Damodaran , S. Venkatachalapathy , F. Alisafaei , A. V. Radhakrishnan , D. Sharma Jokhun , V. B. Shenoy , and G. V. Shivashankar , Mol. Biol. Cell 29, 3039 (2018). 10.1091/mbc.E18-04-0256 30256731 PMC6333178

[94] K. V. Iyer , A. Taubenberger , S. A. Zeidan , N. A. Dye , S. Eaton , and F. Jülicher , Nat. Commun. 12, 1756 (2021). 10.1038/s41467-021-22010-9 33767161 PMC7994818

[95] A. B. Roberts , J. Zhang , V. R. Singh , M. Nikolić , E. Moeendarbary , R. D. Kamm , P. T. C. So , and G. Scarcelli , J. Biomech. 121, 110400 (2021). 10.1016/j.jbiomech.2021.110400 33882444 PMC8274349

[96] C. M. Denais , R. M. Gilbert , P. Isermann , A. L. McGregor , M. te Lindert , B. Weigelin , P. M. Davidson , P. Friedl , K. Wolf , and J. Lammerding , Science 352, 353–358 (2016). 10.1126/science.aad7297 27013428 PMC4833568

[97] J. Irianto , C. R. Pfeifer , R. R. Bennett , Y. Xia , I. L. Ivanovska , A. J. Liu , R. A. Greenberg , and D. E. Discher , Mol. Biol. Cell 27, 4011 (2016). 10.1091/mbc.E16-06-0428 27798234 PMC5156542

[98] J. Irianto , Y. Xia , C. R. Pfeifer , R. A. Greenberg , and D. E. Discher , Biophys. J. 112, 446 (2017). 10.1016/j.bpj.2016.09.047 28341535 PMC5300774

[99] Y. Xia , I. L. Ivanovska , K. Z. Zhu , L. Smith , J. Irianto , C. R. Pfeifer , C. M. Alvey , J. Z. Ji , D. Z. Liu , S. Cho , R. R. Bennett , A. J. Liu , R. A. Greenberg , and D. E. Discher , J. Cell Biol. 217, 3796 (2018). 10.1083/jcb.201711161 30171044 PMC6219729

[100] C. R. Pfeifer , Y. T. Xia , K. Z. Zhu , D. Z. Liu , J. Irianto , V. M. M. Garcia , L. M. S. Millan , B. Niese , S. Harding , D. Deviri , R. A. Greenberg , and D. E. Discher , Mol. Biol. Cell 29, 1948 (2018). 10.1091/mbc.E18-02-0079 29742017 PMC6232975

[101] Y. T. Xia , C. R. Pfeifer , K. Z. Zhu , J. Irianto , D. Z. Liu , K. Pannell , E. J. Chen , L. J. Dooling , M. P. Tobin , M. Wang , I. L. Ivanovska , L. R. Smith , R. A. Greenberg , and D. E. Discher , J. Cell Biol. 218, 2545 (2019). 10.1083/jcb.201811100 31239284 PMC6683732

[102] S. Cho , M. Vashisth , A. Abbas , S. Majkut , K. Vogel , Y. T. Xia , I. L. Ivanovska , J. Irianto , M. Tewari , K. Z. Zhu , E. D. Tichy , F. Mourkioti , H. Y. Tang , R. A. Greenberg , B. L. Prosser , and D. E. Discher , Dev. Cell 49, 920 (2019). 10.1016/j.devcel.2019.04.020 31105008 PMC6581604

[103] A. J. Earle , T. J. Kirby , G. R. Fedorchak , P. Isermann , J. Patel , S. Iruvanti , S. A. Moore , G. Bonne , L. L. Wallrath , and J. Lammerding , Nat. Mater. 19, 464 (2020). 10.1038/s41563-019-0563-5 31844279 PMC7102937

[104] Q. Zhang , A. C. Tamashunas , A. Agrawal , M. Torbati , A. Katiyar , R. B. Dickinson , J. Lammerding , and T. P. Lele , Mol. Biol. Cell 30, 899 (2019). 10.1091/mbc.E18-09-0604 30566037 PMC6589786

[105] H. Q. Le , S. Ghatak , C. Y. Yeung , F. Tellkamp , C. Günschmann , C. Dieterich , A. Yeroslaviz , B. Habermann , A. Pombo , C. M. Niessen , and S. A. Wickström , Nat. Cell Biol. 18, 864 (2016). 10.1038/ncb3387 27398909

[106] J. S. H. Lee , C. M. Hale , P. Panorchan , S. B. Khatau , J. P. George , Y. Tseng , C. L. Stewart , D. Hodzic , and D. Wirtz , Biophys. J. 93, 2542 (2007). 10.1529/biophysj.106.102426 17631533 PMC1965451

[107] Q. Zhang , V. Narayanan , K. L. Mui , C. S. O'Bryan , R. H. Anderson , K. C. Birendra , J. I. Cabe , K. B. Denis , S. Antoku , K. J. Roux , R. B. Dickinson , T. E. Angelini , G. G. Gundersen , D. E. Conway , and T. P. Lele , Curr. Biol. 29, 2826 (2019). 10.1016/j.cub.2019.07.021 31402305 PMC6736724

[108] T. J. Chancellor , J. Lee , C. K. Thodeti , and T. Lele , Biophys. J. 99, 115 (2010). 10.1016/j.bpj.2010.04.011 20655839 PMC2895377

[109] M. L. Lombardi , D. E. Jaalouk , C. M. Shanahan , B. Burke , K. J. Roux , and J. Lammerding , J. Biol. Chem. 286, 26743 (2011). 10.1074/jbc.M111.233700 21652697 PMC3143636

[110] Y. Hu , I. Kireev , M. Plutz , N. Ashourian , and A. S. Belmont , J. Cell Biol. 185, 87 (2009). 10.1083/jcb.200809196 19349581 PMC2700507

[111] A. Tajik , Y. J. Zhang , F. X. Wei , J. Sun , Q. Jia , W. W. Zhou , R. Singh , N. Khanna , A. S. Belmont , and N. Wang , Nat. Mater. 15, 1287 (2016). 10.1038/nmat4729 27548707 PMC5121013

[112] C. T. Halfmann , R. M. Sears , A. Katiyar , B. W. Busselman , L. K. Aman , Q. Zhang , C. S. O'Bryan , T. E. Angelini , T. P. Lele , and K. J. Roux , J. Cell Biol. 218, 2136 (2019). 10.1083/jcb.201901116 31147383 PMC6605789

[113] R. Foisner and L. Gerace , Cell 73, 1267 (1993). 10.1016/0092-8674(93)90355-T 8324822

[114] D. K. Shumaker , K. K. Lee , Y. C. Tanhehco , R. Craigie , and K. L. Wilson , EMBO J. 20, 1754 (2001). 10.1093/emboj/20.7.1754 11285238 PMC145505

[115] J. Sun , J. Chen , E. Mohagheghian , and N. Wang , Sci. Adv. 6, eaay9095 (2020). 10.1126/sciadv.aay9095 32270037 PMC7112933

[116] A. Belyaeva , S. Venkatachalapathy , M. Nagarajan , G. V. Shivashankar , and C. Uhler , Proc. Natl. Acad. Sci. U. S. A. 114, 13714 (2017). 10.1073/pnas.1708028115 29229825 PMC5748172

[117] W. Huang , H. Hu , Q. Zhang , X. Wu , F. Wei , F. Yang , L. Gan , N. Wang , X. Yang , and A. Y. Guo , Oncogene 38, 6818 (2019). 10.1038/s41388-019-0925-0 31406247 PMC6988105

[118] J. Liu , Y. Tan , H. Zhang , Y. Zhang , P. Xu , J. Chen , Y. C. Poh , K. Tang , N. Wang , and B. Huang , Nat. Mater. 11, 734 (2012). 10.1038/nmat3361 22751180 PMC3405191

[119] J. Lv , Y. Liu , F. Cheng , J. Li , Y. Zhou , T. Zhang , N. Zhou , C. Li , Z. Wang , L. Ma , M. Liu , Q. Zhu , X. Liu , K. Tang , J. Ma , H. Zhang , J. Xie , Y. Fang , H. Zhang , N. Wang , Y. Liu , and B. Huang , EMBO J. 40, e106123 (2021). 10.15252/embj.2020106123 33274785 PMC7809788

[120] K. Tang , L. Zhu , J. Chen , D. Wang , L. Zeng , C. Chen , L. Tang , L. Zhou , K. Wei , Y. Zhou , J. Lv , Y. Liu , H. Zhang , J. Ma , and B. Huang , Cancer Res. 81, 4949 (2021). 34348966 10.1158/0008-5472.CAN-21-0753

[121] Y. Oren , M. Tsabar , M. S. Cuoco , L. Amir-Zilberstein , H. F. Cabanos , J. C. Hütter , B. Hu , P. I. Thakore , M. Tabaka , C. P. Fulco , W. Colgan , B. M. Cuevas , S. A. Hurvitz , D. J. Slamon , A. Deik , K. A. Pierce , C. Clish , A. N. Hata , E. Zaganjor , G. Lahav , K. Politi , J. S. Brugge , and A. Regev , Nature 596, 576–582 (2021). 10.1038/s41586-021-03796-6 34381210 PMC9209846

[122] B. Fabry , G. N. Maksym , J. P. Butler , M. Glogauer , D. Navajas , and J. J. Fredberg , Phys. Rev. Lett. 87, 148102 (2001). 10.1103/PhysRevLett.87.148102 11580676

[123] J. J. Chiu and S. Chien , Physiol. Rev. 91, 327 (2011). 10.1152/physrev.00047.2009 21248169 PMC3844671

[124] F. Chowdhury , B. Huang , and N. Wang , Cytoskeleton 78, 249–276 (2021). 10.1002/cm.21658 33754478 PMC8518377

[125] F. M. Boisvert , S. van Koningsbruggen , J. Navascues , and A. I. Lamond , Nat. Rev. Mol. Cell Biol. 8, 574 (2007). 10.1038/nrm2184 17519961

[126] J. G. Gall , Nat. Rev. Mol. Cell Biol. 4, 975 (2003). 10.1038/nrm1262 14685175

[127] A. A. Hyman , C. A. Weber , and F. Jülicher , Annu. Rev. Cell Dev. Biol. 30, 39–58 (2014). 10.1146/annurev-cellbio-100913-013325 25288112

[128] S. F. Banani , H. O. Lee , A. A. Hyman , and M. K. Rosen , Nat. Rev. Mol. Cell Biol. 18, 285 (2017). 10.1038/nrm.2017.7 28225081 PMC7434221

[129] D. L. J. Lafontaine , J. A. Riback , R. Bascetin , and C. P. Brangwynne , Nat. Rev. Mol. Cell Biol. 22, 165–182 (2021). 10.1038/s41580-020-0272-6 32873929

[130] Y. E. Guo , J. C. Manteiga , J. E. Henninger , B. R. Sabari , A. Dall'Agnese , N. M. Hannett , J. H. Spille , L. K. Afeyan , A. V. Zamudio , K. Shrinivas , B. J. Abraham , A. Boija , T. M. Decker , J. K. Rimel , C. B. Fant , T. I. Lee , I. I. Cisse , P. A. Sharp , D. J. Taatje , and R. A. Young , Nature 572, 543–548 (2019). 10.1038/s41586-019-1464-0 31391587 PMC6706314

[131] A. G. Larson , D. Elnatan , M. M. Keenen , M. J. Trnka , J. B. Johnston , A. L. Burlingame , D. A. Agard , S. Redding , and G. J. Narlikar , Nature 547, 236 (2017). 10.1038/nature22822 28636604 PMC5606208

[132] A. R. Strom , A. V. Emelyanov , M. Mir , D. V. Fyodorov , X. Darzacq , and G. H. Karpen , Nature 547, 241 (2017). 10.1038/nature22989 28636597 PMC6022742

[133] H. Strickfaden , T. O. Tolsma , A. Sharma , D. A. Underhill , J. C. Hansen , and M. J. Hendzel , Cell 183, 1772 (2020). 10.1016/j.cell.2020.11.027 33326747

[134] D. T. Murray , M. Kato , Y. Lin , K. R. Thurber , I. Hung , S. L. McKnight , and R. Tycko , Cell 171, 615 (2017). 10.1016/j.cell.2017.08.048 28942918 PMC5650524

[135] S. E. Farr , E. J. Woods , J. A. Joseph , A. Garaizar , and R. Collepardo-Guevara , Nat. Commun. 12, 2883 (2021). 10.1038/s41467-021-23090-3 34001913 PMC8129070

[136] D. T. McSwiggen , M. Mir , X. Darzacq , and R. Tjian , Genes Dev. 33, 1619 (2019). 10.1101/gad.331520.119 31594803 PMC6942051

[137] Y. C. Poh , S. P. Shevtsov , F. Chowdhury , D. C. Wu , S. Na , M. Dundr , and N. Wang , Nat. Commun. 3, 866 (2012). 10.1038/ncomms1873 22643893 PMC3388544

[138] J. Kim , N. C. Venkata , G. A. H. Gonzalez , N. Khanna , and A. S. Belmont , J. Cell Biol. 219, e201904046 (2020). 10.1083/jcb.201904046 31757787 PMC7039209

[139] P. C. Dingal and D. E. Discher , Biophys. J. 107, 2734 (2014). 10.1016/j.bpj.2014.10.042 25468352 PMC4255197

[140] P. H. Wu , D. R. Aroush , A. Asnacios , W. C. Chen , M. E. Dokukin , B. L. Doss , P. Durand-Smet , A. Ekpenyong , J. Guck , N. V. Guz , P. A. Janmey , J. S. H. Lee , N. M. Moore , A. Ott , Y. C. Poh , R. Ros , M. Sander , I. Sokolov , J. R. Staunton , N. Wang , G. Whyte , and D. Wirtz , Nat. Methods 15, 491 (2018). 10.1038/s41592-018-0015-1 29915189 PMC6582221

